# Humans Can Track But Fail to Predict Accelerating Objects

**DOI:** 10.1523/ENEURO.0185-22.2022

**Published:** 2022-09-09

**Authors:** Philipp Kreyenmeier, Luca Kämmer, Jolande Fooken, Miriam Spering

**Affiliations:** 1 Graduate Program in Neuroscience, University of British Columbia, British Columbia, Canada, V6T 1Z3; 2 Department of Ophthalmology and Visual Sciences, University of British Columbia, British Columbia, Canada, V6T 1Z3; 3 Djavad Mowafaghian Centre for Brain Health, University of British Columbia, British Columbia, Canada, V6T 1Z3; 4 Centre for Neuroscience Studies, Queen’s University, Ontario, Canada, K7L 3N6; 5 Institute for Computing, Information, and Cognitive Systems, University of British Columbia, British Columbia, Canada, V6T 1Z4

**Keywords:** acceleration, eye-hand coordination, manual interception, prediction, saccades, smooth pursuit

## Abstract

Objects in our visual environment often move unpredictably and can suddenly speed up or slow down. The ability to account for acceleration when interacting with moving objects can be critical for survival. Here, we investigate how human observers track an accelerating target with their eyes and predict its time of reappearance after a temporal occlusion by making an interceptive hand movement. Before occlusion, observers smoothly tracked the accelerating target with their eyes. At the time of occlusion, observers made a predictive saccade to the location where they subsequently intercepted the target with a quick pointing movement. We tested how observers integrated target motion information by comparing three alternative models that describe time-to-contact (TTC) based on the (1) final target velocity sample before occlusion, (2) average target velocity before occlusion, or (3) final target velocity and the rate of target acceleration. We show that observers were able to accurately track the accelerating target with visually-guided smooth pursuit eye movements. However, the timing of the predictive saccade and manual interception revealed inability to act on target acceleration when predicting TTC. Instead, interception timing was best described by the final velocity model that relies on extrapolating the last available target velocity sample before occlusion. Moreover, predictive saccades and manual interception showed similar insensitivity to target acceleration and were correlated on a trial-by-trial basis. These findings provide compelling evidence for the failure of integrating target acceleration into predictive models of target motion that drive both interceptive eye and hand movements.

## Significance Statement

Acceleration is an essential feature of most moving objects in our environment, but the human visual system is surprisingly insensitive to acceleration. We investigated observers’ ability to track an accelerating and disappearing target with their eyes and to predict its time of reappearance by making an interceptive hand movement. Despite the ability to accurately track the accelerating target with their eyes, observers failed to consider acceleration when predicting target reappearance, resulting in systematic interception errors. The magnitude of the error can be explained by a model that describes interception timing based on extrapolation of the last available target velocity signal. Observers fail to account for acceleration during target interception and instead update target velocity while the target is visible.

## Introduction

Seeing and perceiving object motion is a vital capability of the primate visual system. Animals hunting for prey or pedestrians crossing a street must be able to act on a target’s speed, direction, and sudden target accelerations or decelerations. Although acceleration is an important feature that describes the behavior of many moving objects, it is well established that the primate perceptual system is largely insensitive to it (Gottsdanker et al., 1961; Calderone and Kaiser, 1989; Werkhoven et al., 1992; Brouwer et al., 2002; Benguigui et al., 2003; Watamaniuk and Heinen, 2003; Mueller et al., 2017). How we incorporate visual acceleration signals into motor commands to interact with moving objects is still not fully understood. Here, we evaluate human observers’ ability to track accelerating targets with their eyes and to predict accelerating target trajectories for manual target interception.

Tracking visual object motion with the eyes and predicting an object’s motion path are two fundamental abilities that rely on decoding visual motion (Fiehler et al., 2019) and can inform interceptive hand movements (Mrotek and Soechting, 2007; Diaz et al., 2013; Mrotek, 2013; Cesqui et al., 2015; Fooken et al., 2016, 2021; De la Malla et al., 2017; Goettker et al., 2019; AJ De Brouwer et al., 2021). Whereas tracking relies heavily on visual signals (Lisberger, 2015), predicting a trajectory also requires memory of previously seen motion (Orban de Xivry et al., 2013; Kowler et al., 2019; Rust and Palmer, 2021).

When tracking moving objects, humans rely on smooth pursuit eye movements, slow rotations of the eyes, to keep the object close to the fovea. Neurons in motion-sensitive extrastriate cortex (area MT) provide the sensory input that drives pursuit. Neurophysiological studies in macaque monkeys found that single neurons in MT are not tuned to a target’s acceleration (Lisberger and Movshon, 1999); instead, acceleration can be decoded indirectly from populations of speed-sensitive MT neurons (Lisberger and Movshon, 1999; Price et al., 2005). These acceleration signals can drive pursuit eye movements in monkeys (Krauzlis and Lisberger, 1994; Churchland and Lisberger, 2001).

Evidence for the use of acceleration signals for human pursuit comes from studies investigating eye movements in response to small perturbations in target velocity (Tavassoli and Ringach, 2010; Brostek et al., 2017) and those investigating eye movements during temporary target occlusion (Bennett et al., 2007). When tracking an accelerating target throughout a brief occlusion period, eye movements (pursuit and saccades) scale with object acceleration in anticipation of target reappearance (Bennett and Barnes, 2006; Bennett et al., 2007; Bennett and Benguigui, 2013). These findings indicate that the pursuit system can extract target acceleration and use this signal to predictively drive continuous tracking eye movements. Importantly, the ability to extract target acceleration signals improves with presentation times (Bennett et al., 2007), indicating that the pursuit system requires a relatively long initial exposure to target acceleration to form an acceleration-based prediction of target motion. However, whether this ability translates to tasks, in which observers predict the reappearance of accelerating objects, is unclear.

Tasks in which observers are asked to intercept accelerating objects with a quick pointing movement, a projectile, or by pressing a button reveal systematic errors, indicating inability to consider target acceleration for action-related motion prediction (Port et al., 1997; Dubrowski and Carnahan, 2002; Benguigui et al., 2003; Benguigui and Bennett, 2010; Bennett and Benguigui, 2013; Brenner and Smeets, 2015; Brenner et al., 2016). These errors were caused by observers’ failure to adjust the timing or position of their hand movement to the target’s acceleration, resulting in interceptions that are too early and ahead of the target when it decelerates, and interceptions that are too late and behind the target when it accelerates.

Whereas eye movements to accelerating targets appear to be responsive to acceleration, hand movements are prone to systematic errors that indicate inability to account for acceleration. Here, we directly compare eye movements and manual interception of accelerating targets in a track-intercept task. We varied the initial target presentation duration and the rates of target acceleration to test observers’ sensitivity to track the accelerating target with their eyes and to predict its time-to-contact (TTC) for manual interception. We hypothesized that observers make systematic interception errors, indicating inability to account for target acceleration. Moreover, we predicted an improved ability to track target acceleration with longer presentation durations, which would also result in better adjustments of interception timing.

## Materials and Methods

**Figure 1.**
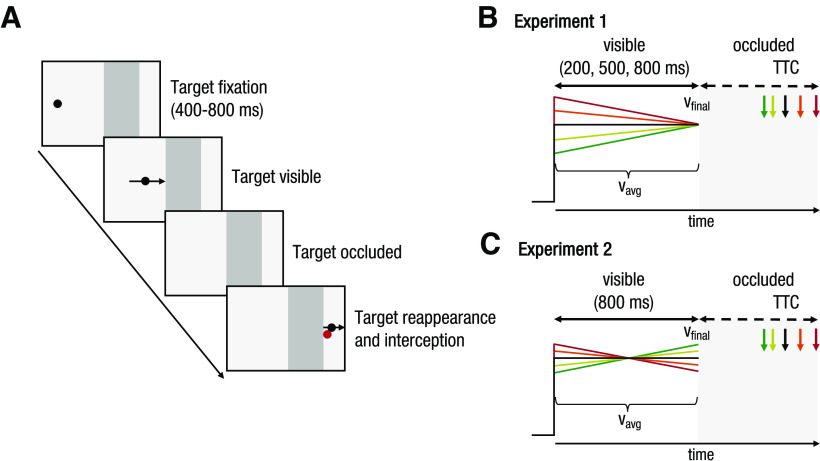
***A***, Task procedure. A black disk moved across a gray monitor background from left to right at a constant rate of acceleration. After an initial period during which the target was visible, it moved behind an occluder of a fixed width (13.4°) and then reappeared. Observers had to estimate the time of reappearance (equivalent to TTC) and intercept the target with a rapid pointing movement of their right index finger (red dot). ***B***, Target parameters in experiment 1. Targets moved with a variable initial velocity (v_init_) and accelerated or decelerated at a constant rate. The initial and average velocities (v_avg_) of the targets were related in such a way with acceleration rate that all targets reached the occluder with the same velocity (v_final_) of 20°/s. ***C***, Target parameters in experiment 2. Initial and final target velocities were related in such a way with acceleration rate that all targets had the same v_avg_ of 20°/s.

We report the results of one main experiment (experiment 1) and one control experiment (experiment 2). Apparatus, procedures, and analyses were identical between both experiments (unless otherwise stated). The critical difference between experiments was that all targets reached the occluder with the exact same final velocity in experiment 1 (Fig. 1*B*), whereas in experiment 2, all targets moved with the exact same average velocity but different final velocities (Fig. 1*C*). The study design and parts of the analyses of experiment 1 were preregistered (https://osf.io/adq9v)

### Observers

#### Experiment 1

We tested 16 human adults (seven females; mean age 26.8 ± 5.1 years, range 19–37 years; including two authors) in this study. All participants had normal or corrected-to-normal visual acuity and reported no history of neurologic, psychiatric, or eye disease. The study protocol was conducted in accordance with the Declaration of Helsinki and was approved by the local Behavioural Research Ethics Board. Participants gave written informed consent before participating and were compensated at the rate of $10/h.

#### Experiment 2

Ten healthy adults were recruited for participation in the control experiments (six females; mean age 28.7 ± 6.7 years, range 21–45 years; four of whom also participated in experiment 1; two authors). Experiment 2 was designed to replicate the model comparison from experiment 1. Specifically, based on experiment 1, we hypothesized that the final velocity model would produce smaller errors, compared with the average velocity model. We used the effect size from experiment 1 (*d *=* *0.86) to determine our sample size of *n *=* *10 using an a priori power analysis in G*Power (Faul et al., 2009; one-sided, paired *t* test; power = 0.80; α = 0.05). Observers gave written informed consent and were compensated at the rate of $10/h.

### Apparatus

Participants performed the task in a dimly lit laboratory, viewing the stimuli binocularly at a distance of 44 cm. A PROPixx video-projector with a resolution of 1280 × 1024 pixels and a refresh rate of 120 Hz (VPixx Technologies) was used to back-project the stimuli onto a 41.8 × 33.4 cm translucent screen. The position of participants’ right eye was recorded using a video-based eye tracker (Eyelink 1000 Tower Mount, SR Research) with a sampling rate of 1 kHz. A combined chin and forehead rest minimized head movements during the experiment. A small magnetic sensor was attached to the tip of participants’ right index finger to record their 3D hand movements with a 3D Guidance trakSTAR (Ascension Technology) at a sampling rate of 120 Hz. The experiment was programmed in MATLAB (MathWorks), using the Psychophysics toolbox (version 3.0.16; Brainard, 1997; Pelli, 1997; Kleiner et al., 2007) and EyeLink toolbox (Cornelissen et al., 2002). Stimulus display and data collection were controlled by a PC (graphics card: NVIDIA GeForce GTX 1060).

### Stimuli and procedure

#### Experiment 1

**Figure 2.**
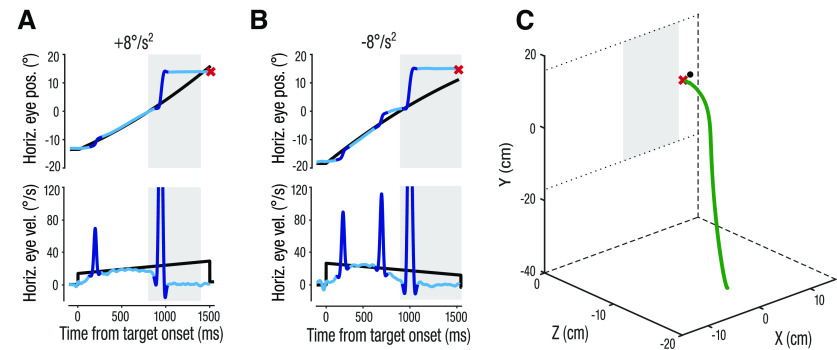
Single trial eye and hand movements from one representative observer. ***A***, ***B***, Two trials with a +8°/s^2^ accelerating target (***A***) or −8°/s^2^ decelerating target (***B***). Light blue traces indicate smooth pursuit components, dark blue traces represent saccades. Upper panels show the horizontal position of the eye (blue) and target (black) locked to target motion onset. The red x represents the interception position and time. Lower panels show horizontal velocity of the eyes and target over time. Gray area represents the time of target occlusion. ***C***, 3D-hand position trace (green) from the same trial as in ***A***. The 2D interception position on the screen is indicated by the red x and the target position at the time of interception in represented by the black disk. The gray area illustrates the position of the occluder on the screen. Dotted lines in the *x-y* plane illustrate the upper and bottom edges of the screen.

Participants viewed and intercepted a small black disk (0.35° in diameter; 6.23 cd/m^2^) that moved across a light gray background (229.8 cd/m^2^) and then disappeared behind an occluder, a gray (181.3 cd/m^2^) bar, that extended 13.4° from the horizontal midline into the right half of the screen. Each trial started with the disk shown on the left side of the screen. Participants had to fixate the target (400–800 ms) and place their index finger on a designated start position on the table located 36 cm below and 23 cm in front of the screen center (Fig. 2*C*). Upon successful fixation, the target started moving to the right, either with a fixed velocity (0°/s^2^) or constantly accelerating at different rates (−8, −4, 4, 8°/s^2^). The target was shown for 200, 500, or 800 ms before occlusion. Participants were instructed to follow the disk closely with their eyes during the initial presentation and to manually intercept the target at the time they expected it to reappear behind the occluder. Target presentation ended either with the time of interception, or 100 ms after target reappearance. Initial target position and velocity were matched for each presentation time and acceleration so that all targets reached the same position and velocity at the time of occlusion. The time of target reappearance (equivalent to TTC) depended on the target’s acceleration rate (−8°/s^2^: 797 ms, −4°/s^2^: 722 ms, 0°/s^2^: 670 ms, +4°/s^2^: 630 ms, +8°/s^2^: 598 ms). Therefore, successful interception required adjusting the timing of manual interception to target acceleration. Observers were instructed to intercept as closely to the border of the occluder as possible at the time they expected the target to reappear. Feedback about the interception performance was provided at the time of interception by showing a red and a black dot, indicating the interception position and the actual target location at the time of interception, respectively. The combination of acceleration and presentation time resulted in 15 experimental conditions, presented in random order within each block of trials. Each participant completed 40 trials per condition, resulting in 600 trials total, presented in eight blocks of 75 trials each. The experiment took ∼90 min.

Although TTC typically refers to the time at which a moving object contacts a secondary, stationary object, we use the term TTC to refer to the time of target reappearance behind the occluder. Furthermore, we use the term TTC_hand_ to refer to observers’ estimate of target reappearance as indicated by the time of manual interception.

#### Experiment 2

Stimuli and procedure were the same as in experiment 1 with the following exceptions. (1) Only the 800-ms condition was tested in experiment 2. (2) All targets moved with the same average velocity but reached the occluder with different final velocities (Fig. 1*C*), yielding occlusion times (TTC) of 1070, 797, 670, 588, and 529 ms for the −8, −4, 0, +4, and +8°/s^2^ acceleration conditions, respectively.

### Eye and hand movement recordings and analyses

The data were preprocessed offline using custom-made routines in MATLAB. Eye velocity and acceleration were calculated as the first and second derivatives of the eye position signals over time. Position and velocity profiles were filtered using a low-pass, second-order Butterworth filter with cutoff frequencies of 15 Hz (position) and 30 Hz (velocity). Saccades were detected when five consecutive frames exceeded a fixed velocity criterion of 30°/s; saccade onsets and offsets were then determined as the nearest reversal in the sign of acceleration. For the analyses of the de-saccaded smooth pursuit eye movements, the identified saccades ±25 ms were removed from pursuit velocity traces and replaced by linear extrapolation between the last velocity sample before saccade onset and the first velocity sample after saccade offset (S De Brouwer et al., 2002). Pursuit onset in de-saccaded traces was detected within a 300-ms interval around stimulus motion onset (starting 150 ms before onset) in each individual trace. We first fitted each 2D position trace with a piecewise linear function, consisting of two linear segments and one break point. The least-squares fitting error was then minimized iteratively (using the function lsqnonlin in MATLAB) to identify the best location of the break point, defined as the time of pursuit onset.

The magnetic hand tracker recorded the 2D screen-centered interception position as well as the participant’s hand movement in 3D space. Hand position data were up-sampled to 1 kHz by linear interpolation for precise temporal comparison with eye movement data. Position data were filtered using a second-order Butterworth filter with a cutoff frequency of 15 Hz. Hand latency was computed offline as the first sample that exceeded 5 cm/s following stimulus onset. Hand movement offset was detected online when the finger intercepted the screen (within 0.8 mm from the screen in the *z*-dimension). If no offset was detected online, hand movement offset was detected offline as the maximum hand position in the *z*-dimension.

All trials were manually inspected. We excluded trials with blinks during the task and trials in which the eye tracker lost the signal (experiment 1: 2.9% of trials across participants; experiment 2: 3.9%). Trials were also excluded when observers undershot the right concluder’s border by >3.5° or when no interception was detected within 600 ms of target reappearance (experiment 1: 1.5% of all trials, experiment 2: 2.3%).

### Data analyses

**Figure 3.**
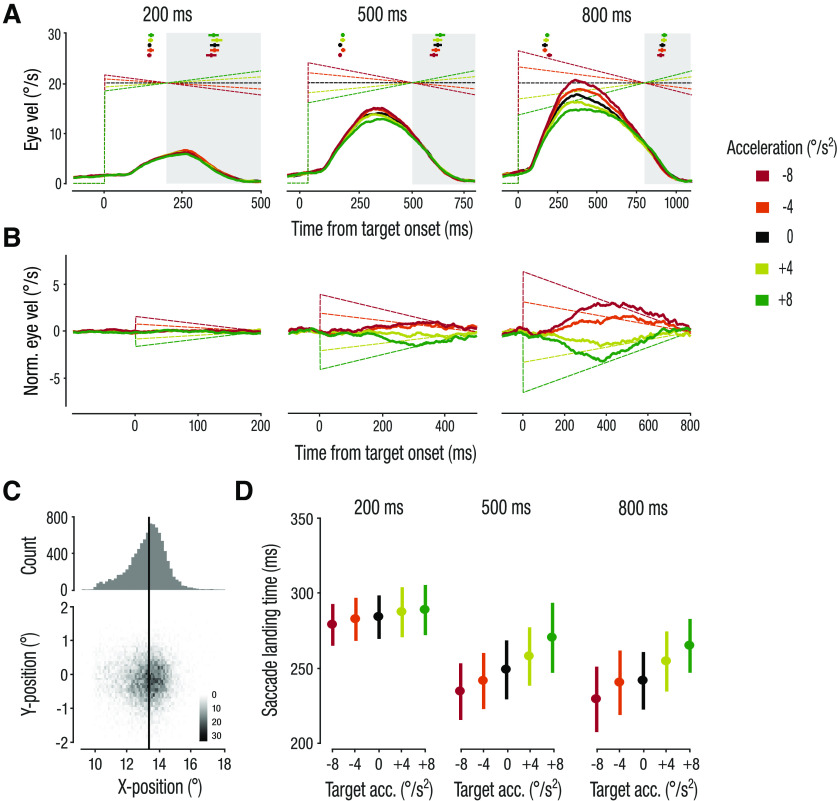
Effect of presentation duration on smooth pursuit and predictive saccades. ***A***, Average de-saccaded smooth pursuit velocities. Squares and error bars at the top show the mean ± 1 SEM of the first catch-up saccade (left) and predictive saccade (right) onsets. Shaded areas indicate occlusion period and dashed lines represent target velocities. ***B***, Normalized pursuit velocity during target presentation. ***C***, Distribution of 2D saccade landing positions. The black line represents the location of target reappearance (i.e., right border of the occluder). ***D***, SLTs. Dots and error bars represent the mean across observers ± 1 SEM.

The primary aim of the current study was to assess whether observers can track and intercept accelerating targets. We assessed observers’ ability to accurately track accelerating targets with smooth pursuit eye movements during the initial target presentation (visually-guided smooth pursuit). We calculated the average de-saccaded pursuit velocity from stimulus or pursuit onset (whichever occurred earlier) and the beginning of target occlusion. Pursuit gain was typically <1 and observers showed anticipatory slowing in the pursuit before target occlusion. To account for these general biases, we normalized pursuit velocity by subtracting the control condition (0°/s^2^) from the experimental conditions (Fig. 3*B*). Following target occlusion, observers typically stopped pursuing the target with smooth pursuit eye movements and used predictive saccades to bring the eyes to the interception location. The primary predictive saccade was determined as the saccade that brought the eye within 3.5° from the occluder’s right border (to allow for systematic undershooting of saccades). The landing time of the eye was determined as the offset time of the predictive saccade. If additional saccades were made to correct the undershooting (2.8% of trials), we used the offset time of the last corrective saccade that was initiated from within the occluder. To assess whether target acceleration was taken into account in manual interception, we calculated the constant interception error as the difference between the time of interception and the veridical time of target reappearance. To assess whether target acceleration caused systematic biases in the timing of the interceptive hand movements, we additionally analyzed the interception time (TTC_hand_). Both saccade landing time (SLT) and TTC_hand_ were calculated relative to target occlusion onset.

#### Model comparison

To further analyze which target features were used to estimate TTC for manual interception, we compared the biases in TTC_hand_ to three different models on how observers might have predicted TTC. First, the final velocity model postulates that observers continuously update target velocity and predict TTC based on the last available velocity sample. Second, the average velocity model assumes that observers base their TTC estimate on the average target velocity. Finally, the acceleration model suggests that observers consider final target velocity and target acceleration for interception.

Because observers showed a general trend to hit the targets too late, we first normalized TTC_hand_ by subtracting the control condition (0°/s^2^) from the experimental conditions. We then calculated targets’ TTC based on three models. For the final velocity model, TTC was calculated as $TTC(x)\;=\;d/{\text{v}}_{final}(x)$, for the average velocity model, TTC was calculated as $TTC(x)\;=\;d/{\text{v}}_{avg}(x)$, for the, and for the acceleration model as $TTC(x)\;=(-{\text{v}}_{final}\;+\sqrt{{\text{v}}_{{final}^{2}}\;+\;2\text{ad}})/\text{a}$. Here, *x* indicates the five different target trajectories, *d* the distance of the occluder, *v_avg_* the average velocity of the target during the initial presentation duration, *v_final_* the final target velocity at the time of occlusion onset, and *a* the rate of target acceleration. We then evaluated the fit of the different model predictions of TTC to our observed TTC_hand_ data by calculating the root-mean-square error (RMSE) for each observer.

To further analyze which target velocity sample best described each observer’s TTC_hand_ bias, we modified the final velocity model to include target velocity as a free parameter, set to optimally predict observers’ TTC_hand_. To this end, we first calculated each observer’s median TTC for each acceleration condition. Next, we predicted each observer’s median TTC based on each time point along each target’s velocity trajectory and determined the time point that produced the smallest RMSE. We coined this time point the hand prediction time. Note, for simplicity, we assumed that observers based their TTC estimate on a single velocity sample. Alternatively, observers might have averaged target velocity over a number of samples.

### Statistical analyses

All statistical analyses were performed in R (version 3.3.2; R Core Team) with an α level of .05. For all outcome variables, we calculated the condition median across trials for each individual observer. The median was used because it is more robust against outliers and skewed distributions. To assess differences in our experimental conditions, we compared the means across subjects using repeated-measures ANOVAs (rmANOVAs) and *post hoc t* tests. Normal distribution of dependent variables for all ANOVAs was confirmed using the Shapiro–Wilk test (all *p *>* *0.14). Violation of sphericity was assessed using the Mauchly’s test and *p*-values were Greenhouse–Geisser corrected in case of significance. To correct the familywise error rate in multiway ANOVA, we applied a sequential Bonferroni procedure to all multiway ANOVA (Cramer et al., 2016). In case of significant interaction effects, we ran follow-up, one-way ANOVA with Bonferroni corrections. Bonferroni corrections were also applied to any pairwise *post hoc* comparisons. To investigate whether SLT predicted TTC_hand_ on a trial-by-trial basis, we ran a linear mixed model with random intercepts and slopes between SLT and TTC_hand_ per observer and SLT, presentation duration, and acceleration as fixed effects (using the functions *mixed* and *nice* of the R package *afex*; Singmann et al., 2021):

$${\text{TTC}}_{\text{hand}}∼\text{ SLT}*\text{presentation duration}*\text{acceleration}+\left(\text{1}+\text{SLT }|\text{ observer}\right).$$

## Results

Observers performed a track-intercept task in which they viewed a moving disk that disappeared behind an occluder after a presentation time of 200, 500, or 800 ms and then reappeared for 100 ms (Fig. 1*A*). We instructed observers to intercept the target at the estimated time of reappearance (equivalent to TTC) with a quick pointing movement of their right index finger. In each trial, the target moved along a horizontal linear path either at a constant velocity (no acceleration, 0°/s^2^: control condition) or at a constant rate of velocity change (deceleration: −8 or −4°/s^2^; acceleration: +4 or +8°/s^2^).

We analyzed the eye and hand movement data in three parts. First, we investigated visually-guided smooth pursuit and predictive eye movements in response to accelerating targets. We asked whether target acceleration was reflected in the visually-guided eye movement response during visible target presentation, and in the timing of the predictive eye movement response during occlusion. Second, we assessed the effect of target acceleration on the timing error of the interceptive hand movement. These two analysis components are congruent with the preregistered analysis plan for this study. Third, we compared the performance of three models predicting TTC based on different target signals to observers’ hand movement data. This exploratory model comparison was confirmed in a control experiment, in which all targets moved with the same average velocity, but with different final velocities (experiment 2). We restricted the exploratory model comparison to the 800-ms conditions, because it yielded the most reliable biases in both eye and hand movements and allowed for the longest integration of the changing target velocities over time.

### Target presentation duration affects ability to track but not to predict accelerating targets

Observers tracked the target with a combination of smooth pursuit and saccadic eye movements during the initial period, in which the target was visible. Two example trials, in which the target either accelerated (Fig. 2*A*) or decelerated (Fig. 2*B*) show typical eye movement position and velocity during the task. After pursuit initiation, an initial catch-up saccade aligned the eyes with the target and was typically followed by a period of closed-loop smooth pursuit, during which eye velocity matched the continuously changing target velocity. In some trials, smooth pursuit was supported by additional catch-up saccades (Fig. 2*B*). Around the time of target occlusion, observers stopped smoothly tracking the target and pursuit velocity decreased to 0°/s. Observers then made a distinctly identifiable predictive saccade of relatively large amplitude to the right border of the occluder, where they then intercepted the target with a pointing movement of their right index finger (see 3D hand trajectory from a single trial in Fig. 2*C*). To investigate how finely-tuned these different eye movement responses are to target acceleration, we analyzed the effect of presentation duration on the ability to smoothly track the visible, accelerating target and to predict its time of reappearance (TTC) with a predictive saccade.

Observers’ ability to accurately and smoothly track the accelerating target increased with increasing presentation duration, here taken as a direct measure of the availability of target motion signals (Fig. 3*A*,*B*). Whereas no difference in average pursuit velocity was observed in the shortest presentation duration (200 ms), average pursuit velocity sensitively reflected different target velocities for both longer presentation durations (500, 800 ms; Fig. 3*A*). This observation was confirmed by a 5 (acceleration) × 3 (presentation duration) rmANOVA on average pursuit velocity, yielding a significant acceleration × presentation duration interaction (*F*_(8,120)_ = 18.52; *p *<* *0.001; *η_p_^2^* = 0.55). Both main effects of presentation duration (*F*_(2,30)_ = 184.76; *p *<* *0.001; *η_p_^2^* = 0.92) and target acceleration (*F*_(4,60)_ = 40.75; *p *<* *0.001; *η_p_^2^* = 0.73) reached significance. We performed follow-up, one-way rmANOVA with factor acceleration for each presentation duration and found a significant main effect of acceleration in both the 500- and 800-ms conditions (*F*_(4,60)_ = 8.31; *p *<* *0.001; *η_p_^2^* = 0.36 and *F*_(4,60)_ = 50.08; *p *<* *0.001; *η_p_^2^* = 0.77, respectively), but not in the 200-ms condition (*F*_(4,60)_ = 1.05; *p *=* *1; *η_p_^2^* = 0.07).

We next asked whether observers continuously tracked the changing target velocity over time. To this end we normalized pursuit velocity traces in the acceleration conditions relative to the control condition (0°/s^2^) for each observer. This additional analysis accounts for imperfect pursuit velocity gain and individual differences.

The normalized pursuit velocity over time revealed how closely observers’ pursuit velocity matched the continuously changing target velocity for the two longer presentation durations (Fig. 3*B*). After the first catch-up saccade, the eye continuously accelerated in response to accelerating targets and decelerated in response to decelerating targets. These findings show that visually-guided pursuit closely matches the continuously changing target velocity, and that these effects are amplified with longer presentation duration where differences in target velocities were more pronounced. In contrast, pursuit velocity was similar across target accelerations in the 200-ms condition. This similarity might have been because of the small differences in the velocity of a target shown only very briefly.

Next, we asked whether the ability to accurately track the accelerating targets with increased presentation duration also affected the ability to predict its reappearance (TTC) with a predictive saccade. Predictive saccades are typically made several hundred milliseconds before target reappearance or interception. Because their timing is tuned to expectations of target motion (Diaz et al., 2013) and reflect decision outcomes in manual interception (Fooken and Spering, 2020), they can provide a sensitive indicator of target motion prediction. Although observers were not instructed to make such a predictive saccade, it was clearly identifiable in virtually every trial (>99% of all trials). These saccades were initiated on average 137 ms (±71 ms; mean ± 1 SD across observers; Fig. 3*A*) after occlusion onset and landed clustered around the right border of the occluder (Fig. 3*C*) ∼262 ms (±70 ms) after occlusion onset. In most trials, observers made one large predictive saccade with an average amplitude of 12.1° (±0.8°) across observers and trials, and this average amplitude was similar across acceleration conditions. On average, saccades were initiated 1.2° (±0.8°) from within the occluder and landed close to the occluder’s right border at 13.2° (±0.3°; Figs. 2*A*,*B*, 3*C*, upper panels). In trials where a second predictive saccade was made to correct for undershooting, initiation and landing time of the first predictive saccade were substantially later (319 ± 94 and 414 ± 93 ms, respectively) than in trials with only one predictive saccade.

Interestingly, the landing time of predictive saccades (SLT) did not scale with target acceleration, i.e., the eye did not land earlier at the border of the occluder for accelerating targets and did not land later for decelerating targets. Instead, the SLT showed a consistent bias in the opposite direction, i.e., later for accelerating targets and earlier for decelerating targets. This observation was confirmed by a main effect of target acceleration (*F*_(4,60)_ = 8.17; *p *=* *0.008; *η_p_^2^* = 0.35; Fig. 3*D*) in a 5 (acceleration) × 3 (presentation duration) rmANOVA. Note that this small bias was also present in the predictive saccade initiation time (Fig. 3*A*) and was neither caused by differences in saccade duration nor in amplitude across acceleration conditions. We also found a main effect of presentation duration on SLT of the predictive saccades (*F*_(2,30)_ = 12.54; *p *=* *0.003; *η_p_^2^* = 0.46). Saccades landed later in the 200-ms condition, compared with the other presentation durations (Fig. 3*D*). Despite the short presentation duration of 200 ms, observers typically made one early catch-up saccade followed by a predictive saccade (Fig. 3*A*, upper panel). This often delayed the onset of the predictive saccade and observers tracked the target until shortly after target occlusion onset. In addition, in a subset of trials (22.7%) in the 200-ms condition, observers only made one saccade. These saccades had slightly larger amplitudes, which might have contributed to the later SLTs in the 200-ms condition. There was no significant interaction between target acceleration and presentation duration (*F*_(8,120)_ = 1.31; *p *=* *0.284; *η_p_^2^* = 0.08). To further analyze which acceleration conditions were significantly different from one another, we performed pairwise comparisons after averaging SLTs across presentation durations. *Post hoc t* tests revealed significant differences between the −8°/s^2^ and the +4°/s^2^ as well as the +8°/s^2^ conditions (*t*_(15)_ = −4.04; *p *=* *0.01 and *t*_(15)_ = −3.35; *p *=* *0.044, respectively), between the −4°/s^2^ and +4°/s^2^ (*t*_(15)_ = −3.90; *p *=* *0.001), and between the 0°/s^2^ and +4°/s^2^ (*t*_(15)_ = −3.29; *p *=* *0.050) acceleration conditions. All other comparisons did not reach significance (all *p *>* *0.10).

Taken together, these findings show that predictive saccades appear to follow a bias in the opposite direction to what we would expect if target acceleration was used to estimate TTC. Moreover, the landing time of predictive saccades was relatively less affected by the presentation duration of the target than what was observed for visually-guided pursuit. Next, we asked whether observers considered target acceleration to time their interceptive hand movement.

### Target acceleration causes systematic manual interception errors

**Figure 4.**
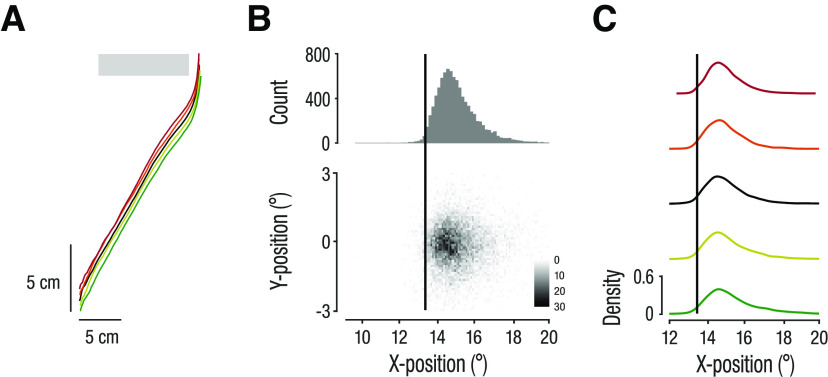
Hand movement paths and interception position. ***A***, Top view of the hand movement paths in the *x-z* plane. The gray bar illustrates the x-position of the occluder on the screen. Hand movement paths were shifted along the *z*-axis for better visibility. ***B***, Distribution of 2D interception positions. Histogram shows the distribution of horizontal interception positions. ***C***, Kernel density plots of horizontal interception positions for the different target acceleration condition.

**Figure 5.**
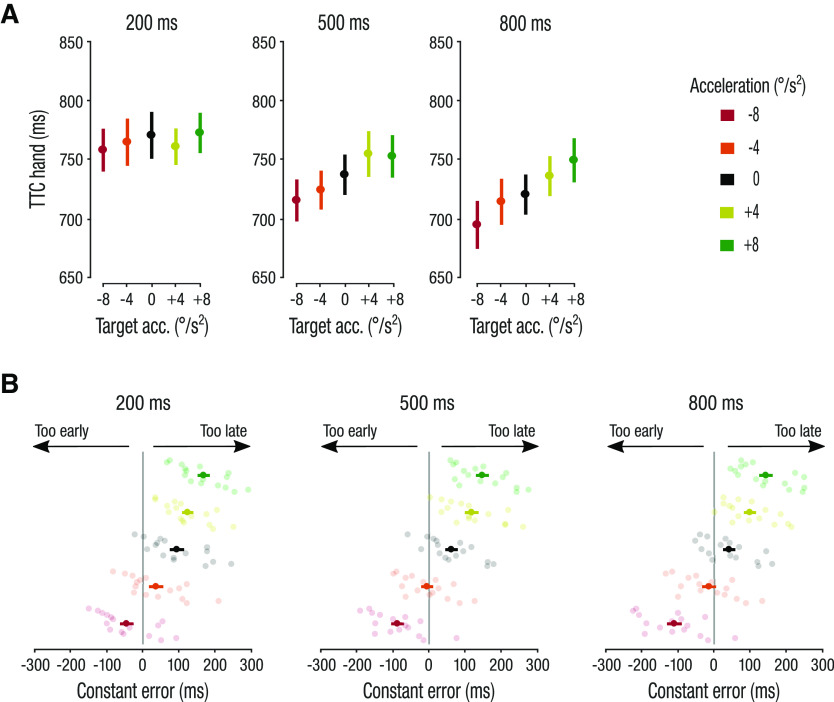
Manual interception time. ***A***, Mean TTC_hand_ (interception time relative to occlusion onset). ***B***, Mean and individual observers’ constant interception errors. Semi-transparent dots represent individual observers’ median performance. Negative values indicate interceptions that occurred before the target reached the end of the occluder (too early), and positive values indicate interceptions occurring after the target reached the end of the occluder (too late). Error bars represent ± 1 SEM.

To intercept the target on the screen, observers moved their hand from the designated start position on the table to the screen (Fig. 2*C*). On average, hand movement paths did not differ between the different target acceleration conditions (Fig. 4*A*). Because of the long occlusion times, hand movements were largely executed during the occlusion and were thus not systematically corrected mid-flight. As instructed, observers intercepted the target close to the occluder’s right border (mean distance 1.7° ± 1.1°; Fig. 4*B*). Distributions of horizontal interception positions largely overlapped across target acceleration conditions (Fig. 4*C*). Yet, there was a small tendency to hit further to the right for accelerating targets (main effect of acceleration on horizontal interception position: *F*_(4,60)_ = 9.21; *p *= 0.006; *η_p_^2^ =* 0.38). This effect was primarily driven by the +8°/s^2^ condition because targets often reappeared before interception in this condition (Fig. 5*B*, green data points). In these trials, observers tended to adjust the interception position, resulting in a more skewed distribution for the +8°/s^2^ condition (Fig. 4*C*, bottom panel).

In parallel to investigating effects of acceleration on the predictive saccade, we next analyzed the effect of target acceleration on the timing of the interceptive hand movement (TTC_hand_). If observers accounted for target acceleration when timing their hand movement, we would expect them to intercept earlier for accelerating targets and later for decelerating targets, relative to the zero-acceleration (control) condition. In contrast to this hypothesis, we found that observers intercepted later in response to target acceleration and earlier for deceleration (main effect of acceleration: *F*_(4,60)_ = 10.80; *p *=* *0.002; *η_p_^2^* = 0.42; Fig. 5*A*). This effect was more pronounced in the 500- and 800-ms presentation duration conditions, confirmed by a significant acceleration × presentation duration interaction (*F*_(8,120)_ = 3.15; *p *=* *0.037; *η_p_^2^* = 0.17). Accordingly, main effects of acceleration were found for both the 500-ms (follow-up, one-way rmANOVA: *F*_(4,60)_ = 10.61; *p *<* *0.001; *η_p_^2^* = 0.41) and 800-ms (*F*_(4,60)_ = 5.94; *p *=* *0.048; *η_p_^2^* = 0.28) conditions but not for the 200-ms condition (*F*_(4,60)_ = 2.62; *p *=* *0.13; *η_p_^2^* = 0.15).

Observers also tended to hit the targets later in the 200-ms condition compared with the 500- and 800-ms conditions, as indicated by a main effect of presentation duration (*F*_(2,30)_ = 16.21; *p *<* *0.001; *η_p_^2^* = 0.52).

The failure to take acceleration into account to time the interceptive hand movement resulted in systematic temporal constant interception errors. Relative to target reappearance, observers intercepted too late (i.e., the target had already reappeared) for accelerating targets and too early for decelerating targets (i.e., the target had not yet reappeared). The main effect of target acceleration on the constant interception error was significant (*F*_(4,60)_ = 492.61; *p *<* *0.001; *η_p_^2^* = 0.97; Fig. 5*B*). We also observed a main effect of presentation duration (*F*_(2,30)_ = 16.47; *p *<* *0.001; *η_p_^2^* = 0.52), which was caused by a general tendency to intercept targets later in the 200-ms condition (see also Fig. 5*A*). Although, there was a significant acceleration × presentation duration interaction term (*F*_(8,120)_ = 3.28; *p *=* *0.032; *η_p_^2^* = 0.18), follow-up, one-way rmANOVA showed main effects of target acceleration on the constant interception error for all three presentation durations (all *p *<* *0.001).

Overall, observers were not able to accurately adjust the timing of their interceptive hand movement (TTC_hand_) to target acceleration, causing systematic constant interception timing errors. Notably, the observed opposite bias in interception time is similar as the bias found for predictive saccades (Fig. 3*D*): the eye lands later and the finger intercepts later for accelerating targets, and both eye and hand intercept earlier for decelerating targets.

**Figure 6.**
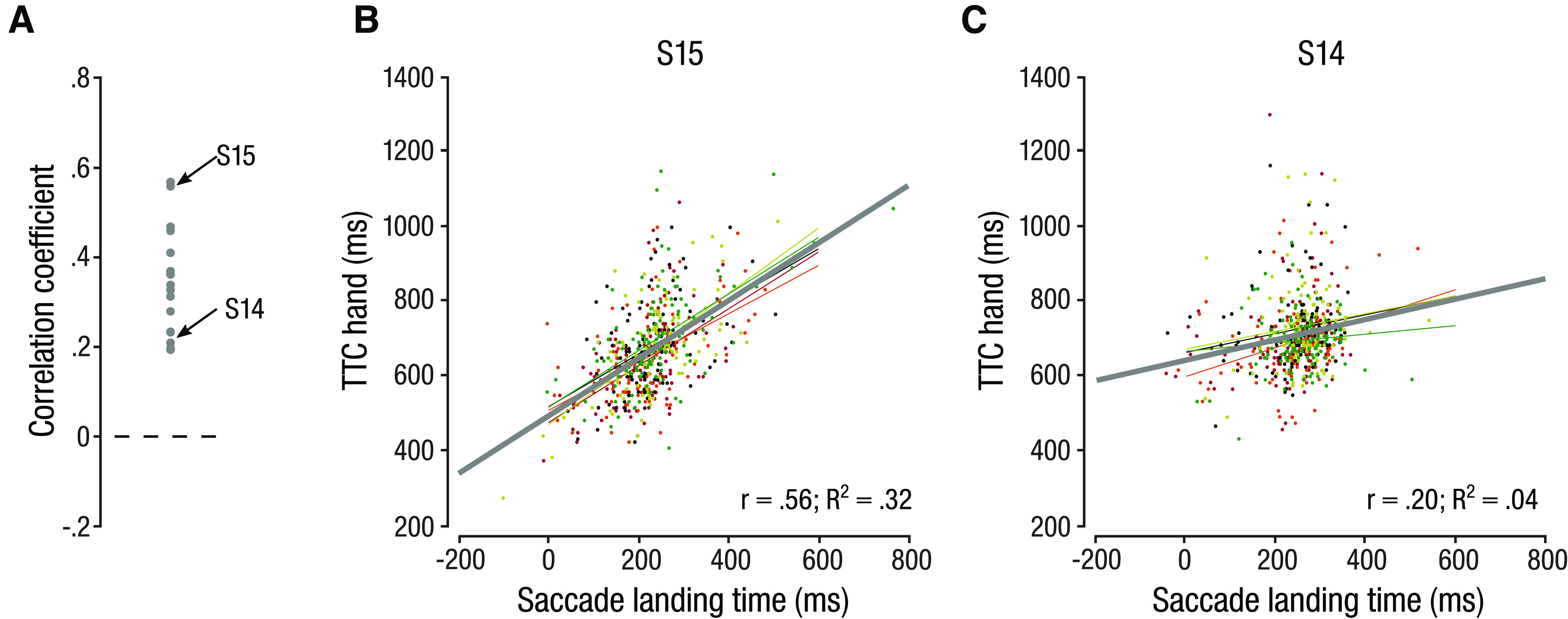
Trial-by-trial correlation across acceleration conditions and presentation durations between SLT and TTC_hand_. ***A***, Distribution of individual correlation coefficients. ***B***, ***C***, Scatterplot and trend lines of the trial-by-trial correlation for an observer with a strong (***B***) and for an observer with a weak (***C***) correlation between SLT and TTC_hand_. Dots represent individual trials. Thin lines represent trend lines for the different acceleration conditions, thick line shows the trend across conditions.

We found that interception time (TTC_hand_) and SLT followed strikingly similar biases opposite to what we would expect if observers accounted for target acceleration. Given that the eye landed at the location of subsequent target reappearance several hundred milliseconds before the hand, we next asked whether SLT was a predictor of TTC_hand_ on a trial-by-trial basis. Trial-by-trial correlations imply a similarity in the trial-based variability between eye and hand movements and are interpreted as evidence for common information sources in the signals that drive eye and hand movements (Sailer et al., 2000). Using a linear mixed model, we found that SLT indeed significantly predicted TTC_hand_ on a trial-by-trial basis (*b *=* *0.351; *F*_(1,15.73)_ = 66.96; *p *<* *0.001). Note, however, that the relation between SLT and TTC_hand_ varied substantially across observers, indicated by individual trial-by-trial Person’s correlations ranging from *r *=* *0.20 to *r *=* *0.57 (Fig. 6*A–C*).

Our findings of similar biases in predictive saccades and prediction-based interceptive hand movements and of a medium trial-by-trial correlation between saccade and hand movement timing suggest that both systems relied on similar motion prediction. Notably, despite the ability to closely track accelerating objects with visually-guided pursuit, predictive eye and hand movements appear to be insensitive to target acceleration, raising the question which target features were used to predict TTC for manual interception.

### Which target features determine TTC predictions?

**Figure 7.**
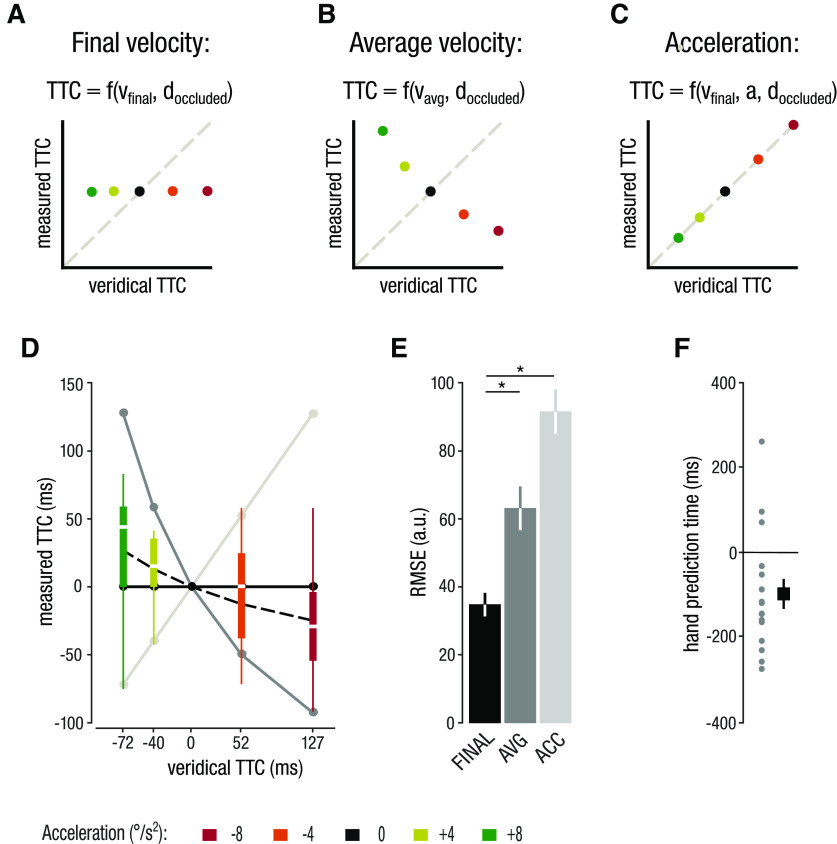
Model comparison. ***A–C***, Three competing models of how observers might predict TTC for predictive eye and interceptive hand movements. ***A***, The final velocity model postulates that observers predict TTC based on v_final_ (identical for all targets in our design, hence predicting a fixed TTC). ***B***, The average velocity model predicts interception timing based on the average target velocity before occlusion, yielding a negative correlation between veridical and measured TTC. ***C***, The acceleration model suggests that observers use target acceleration for interception and predicts the veridical TTC. ***D***, Comparison of model predictions and measured TTC_hand_ data. Dashed line shows the TTC prediction based on the mean hand prediction times. ***E***, Root mean squared errors for the competing models on TTC_hand_. ***F***, Individual and mean hand prediction times. Asterisks represent significant differences (*p* < 0.05).

The observed constant interception errors and the systematic biases in TTC_hand_ (later interception for accelerating targets and earlier interception for decelerating targets) suggest that observers were not able to correctly adjust their interception timing according to target acceleration. We next asked which target features observers used instead to estimate TTC for manual interception. We compared three competing models, describing which target motion signals observers might have used to estimate TTC of accelerating targets (Bennett et al., 2007; Heinen, 2007; Fig. 7*A–C*).

As one possibility, observers could continuously update target velocity and estimate TTC based on the last available target velocity sample before occlusion (final velocity model; Fig. 7*A*). Alternatively, observers might use the average velocity during the visible period to estimate target reappearance (average velocity model; Fig. 7*B*). Finally, if observers indeed considered target acceleration, we would predict accurate scaling of the estimated TTC with target acceleration (acceleration model; Fig. 7*C*). We compared the TTC predictions of the different models to the observed biases in TTC_hand_ (Fig. 7*D*) and quantified the performance of each model by calculating the RMSE for each observer.

Using the acceleration model to predict TTC data in eye and hand interception confirms that interception does not use acceleration (Fig. 7*D*, bright gray line). Although the average velocity model captures the small, reversed trend we observed in TTC_hand_ (Fig. 7*D*, dark gray line), this model performs poorly at predicting the measured TTC_hand_. The final velocity model produced the lowest RMSEs (Fig. 7*D*, black line). These observations were confirmed statistically by a main effect of *model* on RMSEs in a one-way rmANOVA (*F*_(2,30)_ = 18.00; *p *<* *0.001; *η_p_^2^* = 0.55) and *post hoc* pairwise comparisons. These showed that the final velocity model produced significantly lower RMSEs compared with the acceleration (*t*_(15)_ = 9.64; *p *<* *0.001; *d *=* *2.41) and average velocity models (*t*_(15)_ = 3.46; *p *=* *0.011; *d *=* *0.86; Fig. 7*E*).

The final velocity model predicts that observers rely on the last available velocity sample. To pinpoint the approximate time sample observers relied on when estimating TTC, we determined the time point along each target’s velocity trajectory that best accounted for each observer’s bias in TTC_hand_. We termed this the hand prediction time. A negative value indicates that the observer based the TTC prediction on a velocity sample before occlusion, whereas a positive value would indicate that the TTC prediction was based on a partial extrapolation of the veridical target trajectory during occlusion. We found an average hand prediction time of −96.4 ms (Fig. 7*F*), which captures the small opposite bias we observed in TTC_hand_ (Fig. 7*D*, dashed in line).

### Final velocity model prediction generalizes to different target configurations

**Figure 8.**
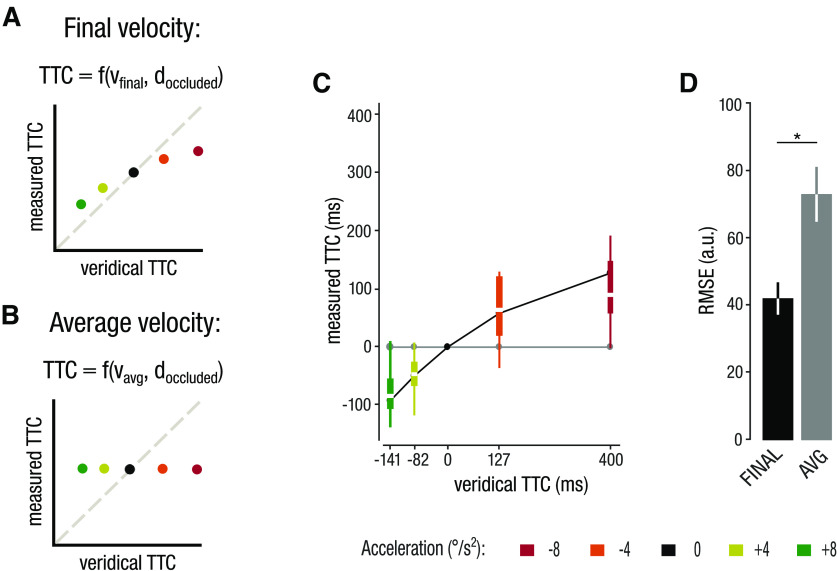
Results from experiment 2. ***A***, ***B***, The model predictions of the final (***A***) and average velocity (***B***) models in experiment 2. ***C***, Comparison of TTC_hand_ to the model predictions. ***E***, Comparison of model fits. Asterisks represent significant differences (*p* < 0.05).

Our model comparison indicates that observers continuously updated their prediction of target velocity until shortly before occlusion. One possible shortcoming of our experimental design was that the target moved at the same final velocity in all acceleration conditions. This might have induced a bias to always hit the target at the same time, favoring the final velocity model. Moreover, our data also showed a small bias toward the average velocity model. We conducted a control experiment to address whether the predictions of the final velocity model hold true when targets moved with different final velocities. In the control experiment, all targets moved with the same average velocities and different final velocities (Fig. 1*C*). Importantly, these target configurations predict the same difference in RMSEs between the average and final velocity models as in experiment 1, allowing us to directly compare the model fits between the two experiments. Note that in contrast to experiment 1, the average velocity model here predicts no TTC adjustments, whereas the final velocity model predicts earlier TTC for accelerating targets and later TTC for decelerating targets (Fig. 8*A*,*B*).

In line with the prediction of the final velocity model, we found that observers intercepted accelerating targets earlier (Fig. 8*C*, negative TTC values) and decelerating targets later (Fig. 8*C*, positive TTC values). This observation was supported by a significant one-sided paired *t* test, testing whether the final velocity model produced significantly lower RMSEs compared with the average velocity model (*t*_(9)_ = 2.12; *p *=* *0.031; *d *=* *0.67; Fig. 8*D*). We also estimated the target velocity sample that best predicted observers’ TTC bias. We found a mean hand prediction time of 65.8 ms before occlusion onset, which was similar but slightly smaller than the hand prediction time in experiment 1. Together, these results replicate our findings from experiment 1 and confirm the use of the final target velocity to predict TTC.

## Discussion

The aim of the current study was to investigate how humans integrate visual motion information to track and predict accelerating objects for manual interception. Our task required observers to track an accelerating target before a temporary occlusion, and to predict the time of target reappearance by making an interceptive hand movement. This hand movement was naturally accompanied by a predictive saccade to the interception position, although no explicit instruction to make such a saccade was given. Our results show that observers were insensitive to target acceleration when predicting future target motion. Neither the timing of the predictive saccade nor the interceptive hand movement (TTC_hand_) scaled with acceleration, resulting in systematic constant interception errors. Inability to account for target acceleration was observed regardless of target presentation duration and of whether observers were able to accurately track the accelerating target before the occlusion. TTC estimates were best described by a model that relied on the final velocity of the target just before occlusion, indicating that observers based their prediction on the memory of the last available velocity signal (first-order motion; Benguigui et al., 2003; Benguigui and Bennett, 2010).

### Different acceleration sensitivity for tracking and predicting?

To successfully interact with a moving object, we must continuously monitor its dynamically-changing motion trajectory. Because of sensorimotor delays, we need to quickly form a prediction of current and future object motion. This form of prediction is also important when we lose sight of an object, or when it is temporarily occluded. Naturally-moving objects do not necessarily move at constant velocity but can suddenly accelerate or decelerate. Forming a prediction that can capture dynamically-changing object motion is therefore an integral part of everyday actions (Zhao and Warren, 2015; Fiehler et al., 2019).

The abilities to track and predict the motion trajectory of objects that move at constant velocity are often closely linked (Makin and Poliakoff, 2011). Accurate tracking of a moving object with smooth pursuit eye movements can enhance temporal (Bennett et al., 2010) and spatial (Spering et al., 2011) predictions of target trajectories. Yet, our results suggest that tracking an accelerating target does not necessarily extend to predicting accelerating objects when aiming to intercept them. Specifically, we show that observers’ eye movements closely matched the velocity profile of accelerating targets for target presentations of longer than 200 ms. However, regardless of how long observers had time to track and potentially integrate acceleration signals, they did not consider target acceleration when timing their manual interception and predictive saccade.

One explanation for the apparent discrepancy between tracking and predicting accelerating targets could be that visually-guided tracking can rely on detecting and updating the changing target velocity over time and might thus not require a direct consideration of the acceleration signal. Conversely, to intercept accelerating targets, observers would need to consider an explicit readout of target acceleration to form a prediction of target motion to overcome sensorimotor delays or a temporary occlusion of the moving object. The finding that acceleration is not used during manual interception suggests that observers continuously update their judgment of target velocity but cannot integrate acceleration signals to inform their prediction. Instead, they predicted TTC based on a velocity sample ∼100 ms before target occlusion, suggesting the use of velocity memory when predicting future target motion (Soechting et al., 2009; Benguigui and Bennett, 2010; Rust and Palmer, 2021). Continuously tracking the changing target velocity with smooth pursuit eye movements might have thus supported manual interception by continuously updating observers’ prediction of target velocity.

Alternatively, tracking and predicting might exhibit different sensitivity to acceleration signals. A possible dissociation in integrating acceleration signals between tracking and interception is congruent with two sets of literature that have typically tested both behaviors, tracking and interception, separately. First, the smooth pursuit system can be sensitive to acceleration signals when probing it with velocity perturbations (Tavassoli and Ringach, 2010; Brostek et al., 2017). Moreover, predictive pursuit during a target’s occlusion period scales with the target’s acceleration (Bennett and Barnes, 2006; Bennett et al., 2007; Bennett and Benguigui, 2013). These findings suggest that the oculomotor system can extract acceleration signals even to predictively drive pursuit. Second, interceptive hand movements are comparatively unresponsive to visual acceleration, reflected in systematic errors when intercepting accelerating targets (Port et al., 1997; Dubrowski and Carnahan, 2002; Benguigui et al., 2003; Brenner and Smeets, 2015; Brenner et al., 2016). These systematic interception errors can be explained by a failure to predict accelerating target motion to overcome sensorimotor delays (Brenner and Smeets, 2015) or temporary target occlusion, in line with our results (see also Reid and Dessing, 2018). Moreover, we showed that systematic interception errors can be described by a model that relied on target velocity just before target occlusion (first-order motion; Benguigui et al., 2003; Benguigui and Bennett, 2010). Together, our results are congruent with the idea that target velocity estimates are continuously updated for both visually-guided pursuit and prediction-guided interception.

### Extending the eye-hand link to prediction-based actions

We observed trial-by-trial correlations between the timing of the predictive saccade and TTC_hand_, extending the known close coupling of eye and hand movements during visually-guided actions (Hayhoe, 2017; AJ De Brouwer et al., 2021) to predictive actions (Binaee and Diaz, 2019). During visually-guided reaching, observers commonly shift their eyes to the reach target before hand movement execution (Ballard et al., 1992; Neggers and Bekkering, 2000; Johansson et al., 2001; Land and Hayhoe, 2001; Horstmann and Hoffmann, 2005; Barany et al., 2020). When intercepting moving targets, observers naturally track the target with their eyes, even when no explicit instruction to do so is given (Mrotek and Soechting, 2007). In interception tasks, eye and hand movement endpoints are also correlated (Kreyenmeier et al., 2017; Li et al., 2018; Fooken et al., 2021). Our results extend these findings in two ways. First, correlations of predictive eye movements and interceptive hand movements reveal that the coordinated control of eye and hand movements also applies to memory-based actions. Second, correlations of temporally-based estimations show that eye and hand movements can be correlated not just in the spatial, but also in the temporal domain. Similarly, previous studies showed that the timing of predictive saccades is finely tuned to stimulus properties (ball speed and elasticity; Diaz et al., 2013) and is a sensitive indicator of decision outcomes in manual interception tasks (Fooken and Spering, 2020). Although the eyes reach the interception location several hundred milliseconds before the hand in our task, the timing of predictive saccades and interceptive hand movements showed strikingly similar biases and were correlated on a trial-by-trial basis. A strong eye-hand link is expected when intercepting targets that move unpredictably and are partially occluded from view (Fooken et al., 2021). If acceleration is indeed not considered in a predictive model of target motion, the extrapolation of accelerating target motion becomes highly inaccurate and observers rely on their eye movements to continuously update their prediction of the target motion (Brenner and Smeets, 2018; De la Malla et al., 2019).

### Assessing model predictions of accelerating motion integration

Given the limited perceptual sensitivity to acceleration, and the lack of acceleration tuning in key motion-sensitive cortical areas (Lisberger and Movshon, 1999; Price et al., 2005) the question arises what information observers rely on when interacting with accelerating objects in everyday life. It is well known that humans use physical laws of motion, such as gravity, which are learned throughout the lifespan, as an implicit prior when interacting with real-world objects (Zago and Lacquaniti, 2005; Jörges and López-Moliner, 2017). For instance, observers are more accurate when tracking and predicting simulated fly balls that move with natural gravity compared with balls that do not (0 g), or that are unnaturally impacted by gravity (2 g; Bosco et al., 2012; Russo et al., 2017). Although naturalistic priors influence ocular and perceptual motion prediction (Delle Monache et al., 2019), other studies have also found that observers assume that targets move with constant velocity when predicting object motion (Jörges et al., 2021).

When intercepting targets that are impacted by arbitrary acceleration, we found that observers make systematic interception errors (Port et al., 1997; Dubrowski and Carnahan, 2002; Benguigui et al., 2003). In situations where the target is not occluded from view and remains visible throughout, observers can minimize these interception errors by continuously adjusting their interceptive hand movement online (Brenner and Smeets, 1997; Reid and Dessing, 2018). Nonetheless, even when intercepting visible accelerating targets, systematic interception errors occur because of sensorimotor delays (Brenner and Smeets, 2015; Brenner et al., 2016). One possibility to compensate for the inability to extrapolate accelerating motion, is to quickly adapt movements, given that sufficient trial repetitions are available (Ruttle et al., 2021). For example, improvements in the ability to manually intercept (Brenner et al., 2016) and predictively pursue (Bennett and Barnes, 2006) accelerating targets after a few (eight to twelve) repetitions of the same acceleration rate have been reported. These findings suggest that observers might be able to form short-term and long-term (naturalistic) priors to counteract the lack of acceleration signal integration.

In conclusion, our study shows that observers failed to use an acceleration-based prediction of the target’s motion to inform manual interception. Instead, the timing of manual interception was best predicted by an extrapolation of target velocity shortly before target occlusion. Systematic interception errors occurred regardless of the target presentation duration and how well observer’s visually-guided eye movements matched the different target velocity profiles. Interestingly, the timing of both predictive eye and interceptive hand movements showed strikingly similar biases and were correlated on a trial-by-trial basis, indicating a strong coupling between both effectors during prediction-guided interception tasks.

## Citation Diversity Statement

Recent work in several fields of science has identified a bias in citation practices such that papers from women and other minority scholars are under-cited relative to the number of such papers in the field (Zurn et al., 2020). Here, we sought to proactively choose references that reflect the diversity of the field in thought, form of contribution, gender, race, ethnicity, and other factors. First, we obtained the predicted gender of the first and last author of each reference by using databases that store the probability of a first name being carried by a woman (Dworkin et al., 2020; Zhou et al., 2020). By this measure (and excluding self-citations to the first and last authors of our current paper), our references contain 12.9% woman (first)/woman (last), 12.26% man/woman, 22.58% woman/man, and 52.25% man/man. This method is limited in that (1) names, pronouns, and social media profiles used to construct the databases may not, in every case, be indicative of gender identity and (2) it cannot account for intersex, nonbinary, or transgender people. Second, we obtained predicted racial/ethnic category of the first and last author of each reference by databases that store the probability of a first and last name being carried by an author of color (Ambekar et al., 2009; Sood and Laohaprapanon, 2018). By this measure (and excluding self-citations), our references contain 9.24% author of color (first)/author of color (last), 19.02% white author/author of color, 16.68% author of color/white author, and 55.06% white author/white author. This method is limited in that (1) names and Florida Voter Data to make the predictions may not be indicative of racial/ethnic identity, and (2) it cannot account for Indigenous and mixed-race authors, or those who may face differential biases because of the ambiguous racialization or ethnicization of their names. We look forward to future work that could help us to better understand how to support equitable practices in science.

## Synthesis

Reviewing Editor: Nicholas J. Priebe, University of Texas at Austin Decisions are customarily a result of the Reviewing Editor and the peer reviewers coming together and discussing their recommendations until a consensus is reached. When revisions are invited, a fact-based synthesis statement explaining their decision and outlining what is needed to prepare a revision will be listed below. The following reviewer(s) agreed to reveal their identity: NONE.

SYNTHESIS

Two reviewers evaluated the manuscript “Humans can track but fail to predict accelerating objects” and both reviewers both reviewers agree that the authors provide evidence that acceleration cues are accessed minimally, if at all, by our eye movement system and when making predictive hand and eye movements. Both reviewers agreed that this revision was a substantial improvement. There was consensus that while this demonstration is important, there remain two areas of concern with the manuscript in its present form. First, questions were raised about the surprising and important finding that saccadic eye movements are influenced by object acceleration. Reviewers would like a finer description of these saccades including their initiation and landing points. Second, the reviewers raised specific concerns about the statistics employed in the manuscript. These issues, and others, are detailed below, which you should find useful in revising your manuscript, if you decide to resubmit it to eNeuro or to another journal.

Reviewer #1

The manuscript is well written and easier to understand compared to the previous version. There are some questions related to the predictive saccade analysis and, in particular, how/when the saccades are produced in the 200ms view time condition. See below for specific questions.

Line 72. I am not sure previous evidence supports that the smooth pursuit system “effectively” extracts acceleration signals. It is clear that these signal are extracted in some form and utilized at some level, but that their contribution to smooth pursuit behavior has been described as small.

Line 134. Do you mean the Psychophysics toolbox?

Line 155. I believe this is the first use of TTC as time-to-contact in the manuscript. Throughout the manuscript, sometimes TTC is used and sometimes TTChand is used. It might be helpful to the reader to more clearly distinguish the differences between the uses. Also I am not sure the term applies sensibly when it is used to describe the time the target is occluded and reappears. Unless I misunderstand the use of the abbreviation.

Line 220. I see that the saccade landing time was determined by the offset time of the predictive saccade, but how variable was the onset time of the saccade? Can you confirm if the onset time of the predictive saccade was used for any of these types of computations? If not can you provide the onset time and variability of the onset time? (or maybe address in comments below.)

Line 221. Could you describe how often multiple predictive saccades were made while the target was occluded and the provide values of the landing times when one saccade was made versus when multiple saccades were made? Were the participants ever still making predictive/corrective saccades when the target reappeared?

Line 318. In the previous review, Reviewer 3 noted that it is difficult to make a conclusion about how quickly smooth pursuit shows sensitivity to different target velocities. (In this experiment the 200 ms view time shows no difference in pursuit velocity magnitude where the longer view times show that pursuit velocity is different in the various target acceleration conditions. However, the 200 ms view time also has very small differences in target velocity such that it might be difficult to show differences in pursuit velocity as well.) Was smooth pursuit gain or smooth pursuit acceleration examined in attempt to clarify the data relative to this confound?

Further, it appears that in the 200 ms view time condition smooth pursuit velocity continues to increase for about 100 ms (estimated from Figure 3A), whereas smooth pursuit velocity is decreasing at the time of the occlusion in both the 500 ms view time and the 800 ms view time conditions (likely in anticipation of the occlusion itself). Thus, pursuit gain is probably showing different patterns at the onset of the occlusion. Could this impact the lack of pursuit velocity different in the 200 ms view tie condition.

I wonder if the fact that the pursuit velocity increases after the occlusion impacts the predictive saccade onset location and onset timing and overall condition performance differences (see next 2 comments).

Line 354. I am struggling to understand the result that saccade landing time differed by target acceleration condition. Previous research has shown that the metrics of saccades of a specified magnitude are rather consistent. Therefore one might expect that a saccade that is made across an occlusion of constant size would have very similar metrics (time needed to complete and speed, etc.) across all conditions. Thus I am rather surprised that differences were found across the various acceleration conditions. This unexpected finding leads me to wonder if the saccades started in a similar location across conditions and if they started at a similar time across conditions. Also it appears that there is a fairly large amount of variability in the saccade initiation time (+/-71 ms; line 346); is this variability consistent across the view time and target acceleration conditions or is it dependent on the condition?

Line 354. I would also like to know which acceleration conditions were different from one another in a post-hoc analysis. In Figure 3D it looks like there is a lot of overlap in the SEM for the different acceleration conditions, even comparing -8 deg/sec/sec and +8 deg/sec/sec.

Line 359. I wonder about the timing of the catch-up saccade due to the onset of target motion (indicated on line 332 that it began about 300 ms after the onset of target motion) and the timing of the predictive saccade to move the eye to the right side of the occlusion border - specifically in the 200 ms view time condition. My concern is two-fold.

Were sometimes these saccades one and the same? If so, then they were likely of a different magnitude as compared to those in the other view time conditions and started at a different time than in the other view time conditions.

Alternatively in the instances that there were two saccades in the 200 ms condition - then according to your description the catch-up saccade began after the target was occluded and the predictive saccade began sometime after that. If during this same timeframe smooth pursuit velocity was increasing, then again the saccade likely begin at a different time and was of a different magnitude compared to the saccades executed in the 500 and 800 ms view time conditions.

Lines 375 and 377. I am confused at why in Line 375 the text indicates that there is no difference across target acceleration conditions, but in Line 377 the text indicates that the +8deg/sec/sec condition was different.

Line 380. Here, the text indicates that participants may have made corrections in the pointing movement (or online adjustments) in the +8deg/sec/sec target acceleration condition because the time-to-contact constant error was 150-200ms (estimated from Figure 5) after the target reappeared and “disappeared again”.

Are there other clues that that the participants made adjustments in the interception position movement? Are there trial-by-trial deviations in the movement paths that results in greater variance along the average path or some other indication?

Line 461. Was the timepoint along the target trajectory that best accounts for the participant’s interception bias determined from trial-by-trial data or by average data?

Lines 483-489. Would a hand prediction time analysis in this control experiment show similar results as for the first experiment? That is, participants used the target velocity about 100ms before the occlusion to predict interception time.

Reviewer #2

Having reviewed the comments and changes to the manuscript, I think the authors have done a good job in addressing my comments and suggestions, including the addition of an extra experiment. I have only minor concerns remaining.

You denote model 3 with ‘target acceleration’, but that is not enough information. It is final velocity plus acceleration (as also shown in line 242). I think it makes sense to explain that the first time this model is referred to (and in the abstract)

Line 34: should this be expanded to explain what happens with occlusion? E.g., adding ‘as long as it is in view’?

It is good that you conducted a power calculation for Experiment 2, but you did not account for the number of statistical tests you would conduct (see below).

Figure 7G is redundant - data is in panel D and curve could be added there?

Should you include a link to the preregistration in the Methods?

Line 325: you present the results of the ANOVA but do not discuss posthoc tests. This concern holds for nearly all ANOVAs in fact.

Line 349-355. This is about landing time. Have you examined the initiation times/saccade durations? I.e., the main effect of target acceleration on landing time could also be a main effect of target acceleration on saccade initiation (with a fixed saccade duration). This could have slightly different theoretical implications. In general, this finding appears to suggest participants were not making an ‘interceptive’ saccade (they indeed are ‘predictive’ as you call them here - in the Discussion you do refer to interceptive eye movements, though). This saccade was not part of the task/required and maybe timing was not an issue for the oculomotor system? Maybe only the landing position was used to guide the finger movement, while temporal information was separately influencing the hand movement system.

Line 356: statement suggests a difference (‘more pronounced’) that is subsequently not supported by the statistics. I think you should just state that there was no significant difference.

Line 398: for the avoidance of doubt (i.e., to emphasize the temporal rather than spatial nature of this DV), should you call this the temporal constant interception error (or ‘constant error of interceptive timing’). Maybe this is just my bias, having mainly worked with spatial errors in recent times...

Line 390. Statement is incomplete because a significant interaction as such does not imply that the effect of acceleration was more pronounced in the 500ms and 800ms conditions. Your description does not refer to any posthoc tests. Also, given the number of effects examined across the Results (at alpha = 0.05), p = 0.037 should not provide great confidence in this effect. Also, are the analyses of TTChand not correlated with the analyses of the constant interception error? The latter only averages out variations in the occlusion duration, but will still show some of the same patterns as TTChand. The statistics support this (for the interaction and effect of presentation duration).

487: You used a one-sided t-test here, but the reasoning for the control experiment does allow for the average velocity model to be a better fit in this set of conditions. You would thus be testing which model is better (2-tailed), not whether the final velocity model is better than the average velocity model (1-tailed). I appreciate that the statistics would not be significant, but that is just a reflection of the data. Your power analyses were based on alpha = 0.05 and did not take into account the multiple analyses you planned to conduct (warranting a reduced alpha-level in the power calculations) - I think this particular analysis may have suffered from this.

Line 537: Is this really a case for ‘the importance of velocity memory’? Maybe I overemphasize the word ‘importance’. This result does not IMPLY the velocity was sampled 100ms before target occlusion. It could just be the rolling average over a ∼200ms window instead. Maybe replace ‘indicating the importance’ with ‘suggesting a role’. Or maybe I am just being picky - feel free to ignore.

Two statistical notes emerged in consultation:

The concerns about the ANOVA stem from the history of reporting multiway ANOVAs with alpha = 0.05 used for each individual effect. In a 2-way full-factorial ANOVA, 3 independent effects are tested (2 main effects and one interaction), which means that the effective chance of a false positive is not 0.05 even within a single ANOVA. Of course, this issue amplifies for higher order ANOVAs (e.g., 7 effects tested in a 3-way ANOVA). This problem is starting to be recognized (https://link.springer.com/article/10.3758/s13423-015-0913-5), but is not yet consistently remedied within the review process of most journals.

There is a similar concern (about multiplicity) that permeates the power calculations.

The model comparison for the extra experiment is performed using a one-tailed test. The authors state that a potential bias in experiment 1 may have favored the final velocity model. As such, experiment 2 was conducted (which removes this potential bias). If one views experiment 2 as the valid model comparison, a 2-tailed test is warranted. If one views experiment 2 as a replication of experiment 1 (in absence of the bias), a one-tailed test would be ok. It is somewhat in the eye of the beholder, but the issue would have been avoided with a higher power (or corrected alpha level) would have been used in the power calculations (the authors at least performed a power calculation, which is of course good). The authors could opt for increasing their sample size, but they would need to take appropriate corrective action with respect to the alpha level (see Strube. 2006, Behavior Research Methods). At the very least, the investigators should clarify which of the aforementioned views they had in designing the study.
